# Resistome, virulome, mobilome, and biosynthetic gene clusters adaptations of *Acinetobacter baumannii* Mexican strains before and during the COVID-19 pandemic: insights from whole-genome sequencing

**DOI:** 10.3389/fpubh.2026.1830880

**Published:** 2026-08-12

**Authors:** Moisés A. Alejo, Miriam Sarahi Lozano Gamboa, Brian Muñoz Gomez, Karla G. Hernández Magro Gil, Rodolfo García-Contreras, Rachel J. Whitaker, Anastasio Palacios Marmolejo, Corina-Diana Ceapă

**Affiliations:** 1 Microbiology Laboratory, Department of Chemistry of Natural Products, Institute of Chemistry, National Autonomous University of Mexico (UNAM), Mexico City, Mexico; 2 Laboratorio Estatal de Salud Pública del Estado de Aguascalientes (LESP), México/Escuela de Medicina Universidad Cuauhtémoc Plantel Aguascalientes, Aguascalientes, Mexico; 3 Departamento de Microbiología y Parasitología, Facultad de Medicina, Universidad Nacional Autónoma de México, Ciudad de México, Mexico; 4 Carl R. Woese Institute for Genomic Biology, University of Illinois Urbana-Champaign, Urbana, IL, United States; 5 Department of Microbiology, University of Illinois Urbana-Champaign, Urbana, IL, United States

**Keywords:** antimicrobial resistance, genomic surveillance, healthcare associated infections, horizontal gene transfer, whole genome sequencing analysis

## Abstract

**Background:**

*Acinetobacter baumannii* is a critical multidrug-resistant pathogen whose genomic landscape in Mexico has been reshaped by the COVID-19 pandemic. While global studies have highlighted distinctive sequence type distributions, systematic analyses in Mexico remain limited.

**Methods:**

We analyzed 194 genomes, including 47 newly sequenced post-COVID isolates (MIQ), alongside 147 publicly available genomes (HPG). Whole-genome sequencing was combined with phylogenetic reconstruction, resistome and virulome profiling based on gene presence and absence, mobilome analysis, and biosynthetic gene cluster (BGC) characterization.

**Results:**

Two major clades dominated by Oxford STs 758, 208, 417, and 369 were identified. Resistome profiling uncovered 128 distinct resistome profiles (combinations of genes) and 44 emerging antimicrobial resistance genes (ARGs), with an increased number of resistance genes in the strains obtained during the pandemic. Virulome analysis revealed enrichment of metabolic adaptation genes (*argG*, *carA*, *ilvC*) in MIQ strains. Mobilome profiling demonstrated enrichment of IS*AbA1* and IS*AbA3* elements, known to mobilize carbapenemase genes. Mobilome profiling demonstrated enrichment of IS*Aba1* and IS*Aba3* elements, including novel associations such as *bla*_OXA-72_ with IS*Aba27* and *bla*_OXA-66_ with IS*Aba1*. BGC analysis showed conserved siderophores involved in virulence, alongside diversification of the secondary metabolite repertoires in MIQ genomes. Additional observations included geographic mixing of clades across Jalisco, Aguascalientes, and Mexico City and referral bias toward carbapenemase-positive isolates. Because post-COVID isolates were enriched for referred high-risk cases, resistance estimates likely reflect a worst-case hospital scenario rather than community prevalence.

**Conclusion:**

The genomic landscape of *A. baumannii* in Mexico has diversified post-COVID, with evidence of inter-regional transmission, virulome expansion, mobilome-driven ARG dissemination, and metabolic adaptation. These findings underscore the urgent need for coordinated genomic surveillance, functional and clinical validation of adaptation signals, and regionally integrated infection control strategies to mitigate resistance trajectories.

## Graphical abstract

**Figure:**
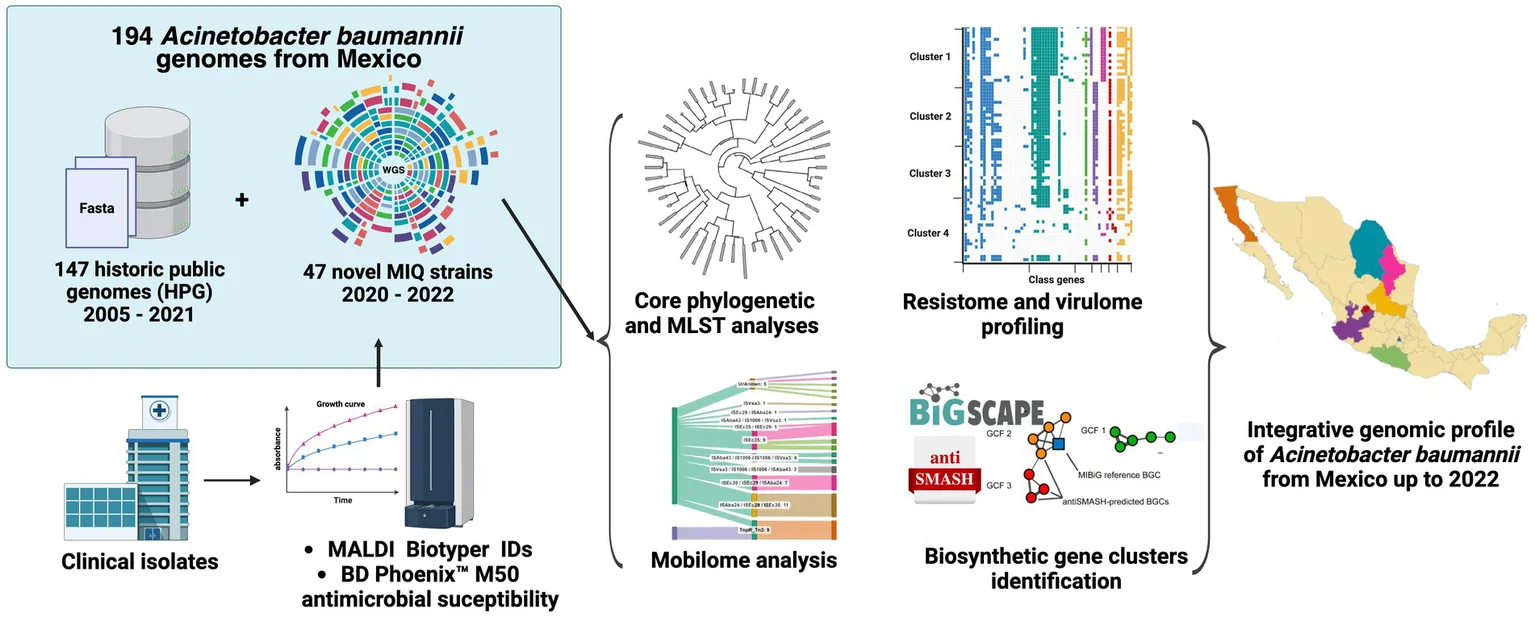
Integrative genomic profiling of *Acinetobacter baumannii* isolates from Mexico (2005–2022). This study combines clinical sampling, phenotypic antimicrobial susceptibility testing, and whole-genome sequencing of 194 *A. baumannii* isolates, including 47 newly sequenced MIQ strains. Comparative genomics reveals structured phylogenetic clades, distinct resistome and virulome profiles, and diverse biosynthetic gene cluster families. Mobile genetic element mapping highlights extensive plasmid–chromosome exchange driving the dissemination of resistance genes across Mexico.

## Introduction

Widespread empirical antibiotic use in COVID-19 patients has led to a significant increase in secondary infections caused by resistant bacteria (1). Among these, *Acinetobacter baumannii* coinfections in ICU settings have surged, frequently exhibiting extensive drug resistance (XDR) or pan-resistance, coupled with high virulence potential, presenting geo-economical specificities (2).

Genomic epidemiology studies from Vietnam, India, and Chile have shown temporal shifts in carbapenem resistance, expansion of high-risk lineages, and extensive genome plasticity in clinical *A. baumannii* populations (3–5). These works underscore how regional healthcare pressures and antimicrobial use shape local clonal structures and resistance trajectories.

Genomic surveillance capacity in Latin America remains highly uneven. Although regional networks such as ReLAVRA+ have strengthened laboratory capacity, few countries conduct systematic national surveillance and reporting, leading to major gaps in publicly available genomic data—often incomplete, missing, or based on isolates collected decades ago (PAHO, 2025).

Recently, global studies indicated that in the pre-pandemic period, Mexico presented sequence type (ST) patterns that differ from neighboring countries (6, 25). Understanding the genomic traits that underpin these phenotypes is essential for effective infection control and the development of therapeutic strategies.

The persistence and pathogenicity of *A. baumannii* are largely attributed to its remarkable ability to evolve and acquire resistance to multiple drugs through diverse mechanisms, including the production of extended-spectrum *β*-lactamases (ESBLs), carbapenemases efflux pumps, and reduced membrane permeability (7, 8). Carbapenem-resistant *A. baumannii* (CRAb) is classified by the WHO as a critical-priority pathogen, reflecting its role as a major medical crisis due to high mortality, extensive drug resistance, and limited therapeutic options (9). Beyond the resistome, the virulome encompasses genes responsible for adherence, biofilm formation, and immune evasion, which contribute to the bacterium’s virulence. The mobilome, consisting of plasmids, transposons, and integrative conjugative elements, plays a pivotal role in the horizontal gene transfer (HGT) of resistance and virulence determinants (6, 23, 25). Additionally, the specialized metabolome, which includes biosynthetic gene clusters (BGCs) responsible for specialized metabolites, may influence bacterial fitness, interspecies competition, and interactions with the host and microbiota (10, 11).

Collectively, these components highlight the complexity of *A. baumannii* adaptability and underscore the urgent need for comprehensive genomic studies to inform effective countermeasures.

In this study, we conducted a comprehensive genomic investigation using whole-genome sequencing (WGS) and *in silico* analyses of clinical isolates collected from Aguascalientes, Mexico City, and neighboring states in Central Mexico during the COVID-19 pandemic and post-pandemic periods (2020–2023) (Graphical Abstract). To provide robust regional and temporal comparisons, we integrated all publicly available genomes characterized across Mexico from the pre-COVID period. This approach provides an integrative and current view of the *A. baumannii* genomic landscape in Mexico, shedding light on the evolution of resistance patterns, virulence factors, mobile genetic elements, and specialized metabolite profiles over time. Our findings aim to support the development of targeted therapies and antimicrobial stewardship programs tailored to the Mexican context.

## Methods

### Ethics and biosafety

This study was approved by the Promotora Médica Aguascalientes S. A. de C. V. Ethics and Biosafety Committee (Approval Nos. 100.cbpma.2025 and 2,937.ceipma.2025), registered with the national regulatory agency COFEPRIS. The protocol encompassed observational, descriptive, and retrospective analyses of multidrug-resistant *A. baumannii*, *Escherichia coli*, and *Klebsiella* spp. isolates collected from hospitalized post-COVID-19 patients between 2020 and 2023. Informed consent was obtained in accordance with Mexican regulations (NOM-012-SSA3-2012), and annual progress reports were submitted to ensure compliance with biosafety and clinical research standards. All procedures adhered to WHO guidelines for antimicrobial resistance surveillance and international ethical frameworks for genomic data sharing (12).

### Clinical samples

Between March 2021 and December 2023, clinical specimens were collected from hospitalized patients at major hospitals in Aguascalientes, including Tercer Milenio General Hospital, General Hospital of Zone No. 1 and No. 2, General Hospital of Pabellón de Arteaga, General Hospital of Rincón de Romos, General Hospital of Calvillo, Women’s Hospital of Aguascalientes, and Centenario Miguel Hidalgo Hospital. Additional isolates were obtained from the Faculty of Medicine, UNAM, and forwarded to the State Public Health Laboratory of Aguascalientes (LESPA).

Isolates were transported under approved biosafety protocols, including triple packaging, cold chain maintenance at 4 °C, and delivery within 48 h. Species-level identification was performed using MALDI-TOF MS (31). Antimicrobial susceptibility testing (AST) was conducted using the BD Phoenix™ M50 system, with interpretation based on CLSI guidelines (CLSI M100, 2023). Extended-spectrum *β*-lactamase (ESBL) and carbapenemase production were confirmed using phenotypic panels (Supplementary Figure 1). Because LESPA receives isolates referred for confirmation by medical practitioners, the dataset is enriched for resistant or clinically severe cases, likely representing a local high-risk subset of hospital circulating pathogens (1).

### Selection of strains for sequencing

Strains were randomly selected from clinical isolates submitted to the LESP, most of which were multidrug-resistant and pan-resistant. The collection reflects this natural bias toward high-risk cases, and final inclusion depended solely on staff time and reagent availability, without any intentional bias toward additional characteristics.

### Whole genome sequencing

Forty-seven clinical strains from patients with lung disease in Central Mexico were cultured on Columbia agar and confirmed by MALDI Biotyper®. Genomic DNA was extracted using the NEB Monarch® kit according to manufacturer’s instructions. Libraries were prepared with the Illumina MiSeq™ DNA Prep kit v3 and sequenced on the Illumina MiniSeq platform using paired-end 2 × 150 bp reads.

Comparative genomic analyses of *A. baumannii* strains were conducted using a standardized bioinformatics workflow designed to ensure reproducibility and robustness. Raw sequencing reads were first subjected to quality control with FastQC v0.11.9, followed by adapter and low-quality base trimming using Trimmomatic v0.39. High-quality reads were assembled *de novo* with SPAdes v3.9.0, and assemblies were annotated using Prokka v1.14.6 and the RAST toolkit implemented in the BV-BRC v3.30.19 platform.

### Public genomes dataset

Historic published genomes (HPG) were retrieved from GenBank by querying for Mexican *A. baumannii* isolates with available metadata on collection year and hospital/state. All genomes meeting these criteria and passing basic quality thresholds (assembly N50 > 50 kb, total length between 3.6–4.3 Mb, and <200 contigs) were included, without further selection, to minimize sampling bias.

*Data Availability* All genomes generated in this study are deposited under BioProject PRJNA1291376 at NCBI. Associated metadata—including collection sites, dates, and sources—are provided in Supplementary Table 1. Draft assemblies available in NCBI were annotated using the NCBI Prokaryotic Genome Annotation Pipeline, while data presented in the text were annotated with SPAdes. Strains are preserved in the Microbiology Laboratory Pathogen Strain Collection, Institute of Chemistry, UNAM, and are available upon request to qualified researchers, especially in support of much needed projects addressing antimicrobial resistance in Latin America (7, 8).

### Phylogenetic analysis

Phylogenetic reconstruction was performed using a combined dataset of newly sequenced MIQ isolates and historic published genomes (HPG) retrieved from GenBank (Supplementary Table 1), thus forming a complete HPG-MIQ dataset used for the following analyses.

Core genome alignments were generated using Roary v3.13.0, and phylogenetic trees were reconstructed with IQ-TREE v2.1.2 under the maximum likelihood framework with 1,000 bootstrap replicates. Assemblies were uniformly annotated with Prokka v1.14.6, and STs were assigned using the PubMLST database.

Core genes were defined as loci present in ≥95% of genomes with ≥90% alignment coverage and ≥90% nucleotide identity. IQ-TREE v2.1.2 was run with ModelFinder, and the best-fit substitution model (e.g., GTR + F + I + G4) was selected according to BIC. Branch support was assessed with 1,000 ultrafast bootstrap replicates.

Metadata (hospital, state, year, Pasteur and Oxford ST schemes) were incorporated into the tree visualization in iTOL v6, allowing contextual interpretation of lineage distribution across clinical and environmental sources.

### MLST analysis

*In silico* multilocus sequence type (MLST) profiles were assigned using the sequences for MLST markers for the Oxford typing scheme (*gltA*, *recA*, *cpn60*, *gyrB*, *gdhB*, *rpoD*, and *gpi*) and Pasteur typing scheme (*gltA*, *recA*, *cpn60*, *fusA*, *pyrG*, *rpoB*, and *rplB*). Sequences were downloaded from the pubMLST database^1^ and then used to extract allele sequences from each of the *A. baumannii* genomes using the BLASTN tool. The extracted sequences were then uploaded to the pubMLST database to assign both existing and novel sequence types.

### Resistome and virulome analysis

For resistome profiling, raw assemblies were screened with the BV-BRC K-mer pipeline, complemented with BLAST searches against the Comprehensive Antibiotic Resistance Database (CARD) and the National Database of Antibiotic Resistant Organisms (NDARO). In addition, we applied SraX, a pipeline optimized for CARD-based annotation (28). Following annotation, redundant entries were removed and homologous genes were grouped to generate a non-redundant resistome dataset suitable for comparative analyses. We defined the ‘core resistome’ as ARGs detected in at least 188/194 genomes (≥97% of the dataset), using a minimum identity of 90% and coverage of 80% in CARD/NDARO-based searches.

We use ‘emerging ARGs’ to refer to resistance genes detected in ≤3 genomes in our dataset, often restricted to post-COVID MIQ isolates or rare lineages, suggesting recent acquisition or limited dissemination.

Resistome diversity was quantified using Bray–Curtis dissimilarity indices, which measure compositional differences in gene content between isolates. The resulting distance matrices were visualized through Principal Coordinates Analysis (PCoA) to identify clustering patterns. To further assess group separation, k-means clustering was applied, and statistical robustness was validated using PERMANOVA (permutational multivariate analysis of variance).

### Mobilome analysis

Mobile genetic elements (MGEs) were systematically characterized for each genome using VRprofile2 (30). For each isolate, we quantified the number and diversity of MGEs. Descriptive statistics were applied to compare mobilome composition between pre-COVID and post-COVID isolates, including median counts per genome and prevalence of specific replicon or transposon families.

To visualize mobilome dynamics, we employed SankeyMATIC to generate Sankey diagrams illustrating gene flow between chromosomes, plasmids, and MGEs.

### Specialized metabolism

All genomes were analyzed with antiSMASH v7.0 (intermediate exhaustiveness). Biosynthetic gene clusters (BGCs) were compared descriptively. Similarity networks of BGCs were constructed with BiG-SCAPE v2.0 and visualized with Cytoscape, referencing MIBiG v3.1 characterized clusters.

## Results

### Phylogenetic landscape

The HPG set included *A. baumannii* strains collected between 2011 and 2018 from diverse Mexican hospitals and institutions (e.g., Hospital Civil de Guadalajara, Instituto Nacional de Cancerología, Hospital Infantil de México Federico Gómez, ISSSTE, and Hospital Universitario de Nuevo León), spanning multiple sequence types (ST1, ST2, ST156, ST422, among others). This integration provided temporal and geographic breadth, enabling direct comparison of post-COVID MIQ isolates with pre-COVID epidemic lineages.

**Figure 1:**
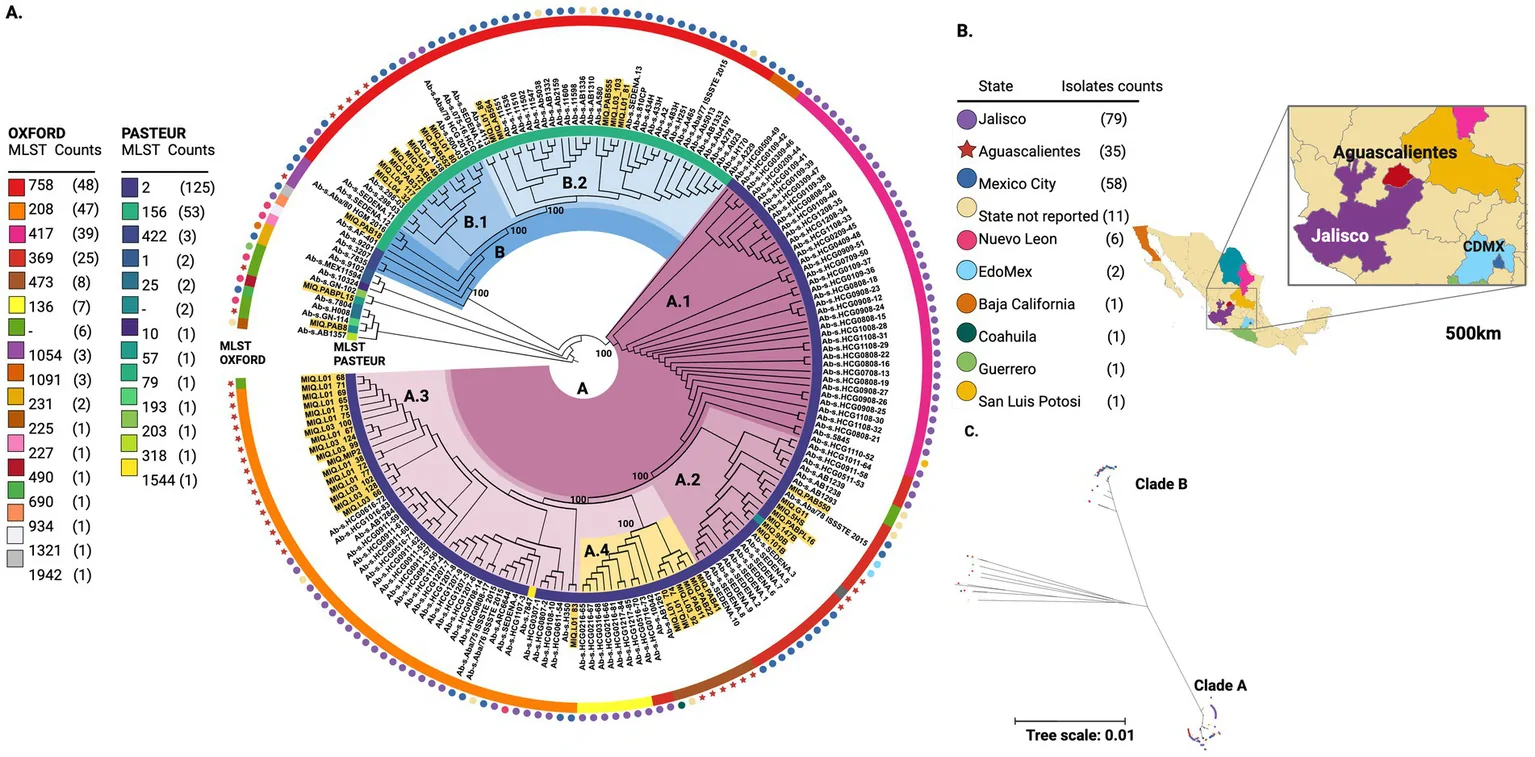
Phylogenetic landscape of *A. baumannii* in Mexico. **(A)** Maximum likelihood phylogenetic tree of 194 genomes, including 47 newly sequenced MIQ isolates and 147 historic published genomes (HPG). Clades A and B are subdivided into subclades (A.1–A.4, B.1, B.2), annotated with Oxford and Pasteur MLST sequence types. The outer ring indicates geographic origin (Jalisco, Aguascalientes, Mexico City, and other states). **(B)** Geographic distribution of isolates across Mexico, with counts per state. **(C)** Simplified tree showing the two dominant clades. Together, these panels illustrate lineage diversity, inter-regional mixing, and the persistence of epidemic clones across time. Created in BioRender. Alejo, A. (2026) https://BioRender.com/8pwzbl4.

Our genomic survey revealed two major clades of *A. baumannii* circulating in Mexico (HPG-MIQ), cutting across geography and time (Figures 1A,B). The phylogenetic trees show that these dominant lineages are not confined to single states but circulate across Jalisco, Aguascalientes, and Mexico City. Clade A was dominated by Pasteur ST2 and ST156, corresponding to international clones IC2 and IC5, respectively, while Clade B was enriched for Oxford ST758, a lineage related to IC2-like backgrounds reported in Latin America. This links the Mexican clades to globally recognized high-risk CRAb lineages.

Based on the canonical *gdhB* allele, the most frequent Oxford STs in our dataset (*n* = 47 MIQ strains) were ST208 (24.0%), ST369 (11.3%), ST417 (19.1%), ST758 (26.0%), and ST136 (3.4%). Post-COVID isolates introduced rare and previously unreported sequence types, signaling new epidemiological trajectories (Figure 1). Within our MIQ collection, 6.3% could not be typed, and rare types such as STs 1, 321, 490, 227, 934, 1942, 690 and 225 appeared.

Pasteur MLST identified nine sequence types: ST2 (65%), ST156 (27%), ST1, ST25, ST79, ST193, ST203, ST318, ST422, and ST1544. ST2 (IC2) and ST156 (CC79, IC5) were the most prevalent, consistent with prior studies. ST2 was widely distributed across central-western Mexico (Aguascalientes, Jalisco, Mexico City, and sporadically Nuevo León, Coahuila, San Luis Potosí). ST156 grouped isolates from central and western Mexico, suggesting inter-regional dissemination.

Within Clade A, ST417, ST369, and ST208 formed three main subclades that were shared between Jalisco, Aguascalientes, and Mexico City, indicating frequent inter-state circulation rather than state-restricted outbreaks. Clade B, dominated by ST758, showed a similar pattern, with one subclade enriched in Mexico City and another spanning Aguascalientes and Guadalajara.

Three genomes (MIQ.90B, MIQ. PAB3, MIQ. PAB8) could not be assigned to existing Pasteur STs, suggesting novel emerging lineages.

The novel Pasteur-unassigned lineages (e.g., MIQ.90B, MIQ. PAB3, MIQ. PAB8) carried expanded resistomes, including carbapenemase genes (*bla*_OXA-72_ or *bla*_OXA-66_) and multiple aminoglycoside and macrolide resistance determinants. Their virulome content was comparable to dominant ST2/ST156 lineages, suggesting that these emerging STs already harbor clinically relevant resistance and virulence repertoires. The closest relatives of these novel lineages were genomes from Germany and Malaysia, which may reflect either long-range dissemination of high-risk clones or convergent acquisition of similar resistance determinants in distinct genomic backgrounds. Given the limited sampling, we interpret these links as suggestive rather than definitive evidence of global spread.

**Table 1:** Antibiotic resistance genes (ARGs) identified in at least 188 out of 194 *A. baumannii* genomes analyzed (core ARGs).

Protein/complex	Mechanism	Antibiotic class(es)
OXA-106	Antibiotic inactivation enzyme	β-lactams
ANT (3”)-II	Antibiotic inactivation enzyme	Aminoglycosides
16S (G1068A)	Target site mutation (16S rRNA)	Aminoglycosides
OprD	Porin loss (outer membrane)	Carbapenems (especially imipenem)
OprB	Porin loss (outer membrane)	Carbapenems
TolC/OpmH	Outer membrane channel component	Supports multiple-flux systems
*ComplexEmrAB-TolC	Efflux pump	Multidrug (quinolones, dyes, detergents)
*Complex MacAB-TolC	Efflux pump	Macrolides
*Complex AdeABC	Efflux pump	Aminoglycosides, fluoroquinolones, tigecycline
*Complex	Efflux pump	Fluoroquinolones, chloramphenicol
*Complex adeIJK	Efflux pump	Broad-spectrum (less regulated than adeABC)
AbeM	Efflux pump	Fluoroquinolones, aminoglycosides
AbeS	Efflux pump	Biocides, dyes, some β-lactams
MdfA/Cmr	Efflux pump	Chloramphenicol, tetracyclines

For gene families or efflux complexes, at least one representative gene was detected in each genome.

### Resistome profiles

**Figure 2:**
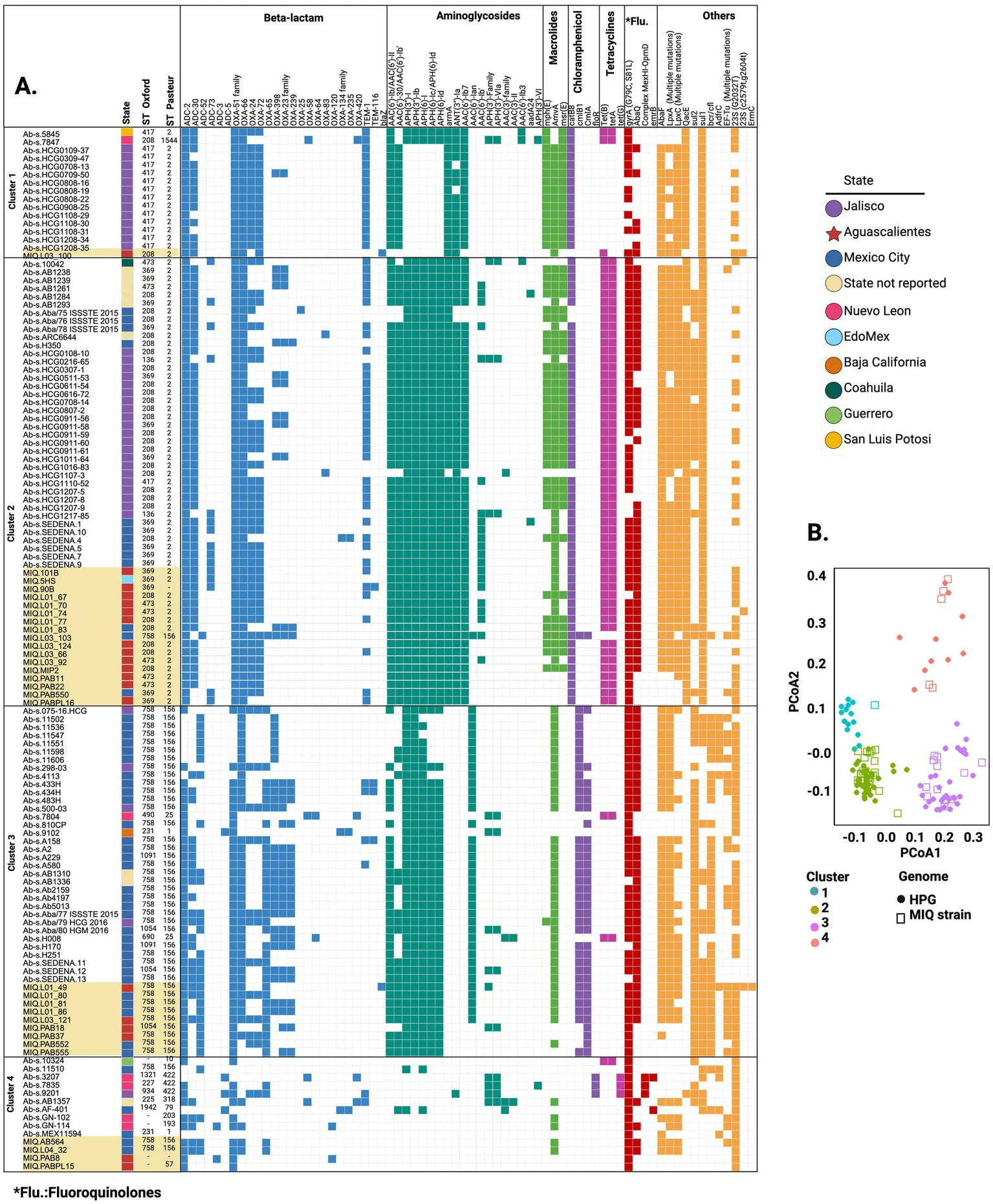
Resistome profiles. **(A)** Heatmap showing antibiotic resistance gene (ARG) presence/absence across MIQ and HPG genomes. Genes are grouped by antibiotic class (*β*‑lactams, aminoglycosides, macrolides, tetracyclines, fluoroquinolones, chloramphenicol, sulfonamides, trimethoprim, oxazolidinones, lipopeptides). Rows represent individual genomes, annotated with sequence type and state of origin. Columns represent ARGs; colored squares indicate presence. The diversity of resistome profiles highlights both conserved core genes and emerging ARGs enriched in post‑COVID isolates. **(B)** Principal Coordinates Analysis (PCoA) plot shows the clustering of the A. baumannii resistome in four groups. Circular markers indicate previously characterized strains, and squares denote MIQ strains. Created in BioRender. Alejo, A. (2026) https://biorender.com/52b2bp1.

Resistome profiling identified 128 distinct gene patterns and 44 emerging ARGs (Figure 2) in the HPG-MIQ dataset.

The core resistome comprises 12 ARGs, including complex and target gene mutation (Table 1). Genes with a unique presence in maximum two genomes (44 genes) (Table 2) were identified in five MIQ strains: MIQ. L01_49, MIQ. L03_100 AB54, MIQ. SA43a, MIQ. PAB3, and MIQ. PABPL15, as well as in some previously characterized strains.

**Table 2:** Antibiotic resistance genes (ARGs) identified in maximum 3 genomes.

Antibiotic class	Function	Gene family	Genome
β-lactams	Antibiotic inactivation	*ADC-76* *OXA-68* *blaZ* *ADC-26* *OXA-96* *OXA-69* *OXA-2* *TEM-157* *TEM-219* *ADC-56* *ADC-6* *OXA-219* *ADC-104* *OXA-78* *ADC-11* *OXA-237* *TEM-171* *ADC-44* *OXA-213* *OXA-332* *ADC-97*	Ab-s.10324 Ab-s.10324 MIQ. L01_49, MIQ. L03_100 AB54 Ab-s.7804 Ab-s.7804 Ab-s.9102 Ab-s.9201 Ab-s. A580 Ab-s. A580 Ab-s. AB1284 Ab-s. AB1357 Ab-s. AB1357 Ab-s. GN-102 Ab-s. GN-102 Ab-s. MEX11594 Ab-s. SEDENA.4 Ab-s. SEDENA.5 MIQ. PAB3 MIQ. PAB3 MIQ. PAB3 MIQ. PABPL15
Aminoglycosides	Antibiotic inactivation	*aad*A5 *ANT (2″)-Ia* *AAC (1)* *aad*A15 *AAC (6′)-Ie-APH (2″)-Ia* *ANT (6)-I* *APH (3′)-IIIa* *sat-4*	Ab-s.9201 Abs.9201 Abs. HCG1107-3 Ab-s. SEDENA.1 MIQ. L01_49 MIQ. L01_49, Abs.9201 MIQ. L01_49 MIQ. L01_49
Daptomycin	Antibiotic target alteration	*cls (ABD31357.1) *rpoC (CAG39569.1) *walK (CAG39047)	MIQ. L03_100 AB54 MIQ. L03_100 AB54 MIQ. L03_100 AB54
Phosphonic acid	Antibiotic inactivation	*fosB*	MIQ. L03_100 AB54
	Antibiotic target alteration	*glpT (CAG39357) *uhpT (CAG39240)	MIQ. L03_100 AB54 MIQ. L03_100 AB54
Multidrug	Efflux pump complex	*mexJK-oprM*	Ab-s.7804
	Antibiotic target alteration	*ermA* *ermC*	MIQ. L03_100 AB54 MIQ. L01_49, MIQ. SA43a
Macrolide	Antibiotic target alteration	*23S(c656t)	MIQ. L03_66
Tetracycline	Antibiotic efflux	*tet (38)*	MIQ. L03_100 AB54
Fluoroquinolone	Antibiotic efflux	*norA*	*MIQ. L03_100 AB54*
Quaternary ammonium compounds	Efflux pump complex	*qacA*	*MIQ. L01_49*
Trimethoprim	Antibiotic inactivation	*dfrC*	*MIQ. L01_49*
Chloramphenicol	Antibiotic inactivation	*cat2*	*Ab-s. AB1357*
Aminocoumarin	Antibiotic target alteration	*gyrB (CAG39033.1)	*MIQ. L03_100 AB54*

* CARD database reported mutation.

*β-lactamases.* A total of 50 β-lactamase genes were identified, including six ADC-type, twelve OXA-type, and four TEM-type variants. Several β-lactamase variants (e.g., ADC-44, ADC-97, OXA-213 family, OXA-332) have not been reported in previous nationwide Mexican surveys of *A. baumannii*, which were dominated by *bla*_OXA-23_ and *bla*_OXA-24/40_-like genes. Their detection here suggests ongoing diversification of β-lactamase repertoires in Mexican hospitals.

ADC-44 and ADC-97 were detected in MIQ. PAB3 and MIQ. PABPL15, respectively. OXA-type β-lactamases, typically associated with *Acinetobacter calcoaceticus* (13), included members of the OXA-213 family in MIQ. PAB3 and MIQ. L04_57, and OXA-332 exclusively in MIQ. PAB3. Other OXA and TEM variants were restricted to previously characterized genomes and were not present in the MIQ genomes.

*Aminoglycosides.* Resistance was mediated by 28 genes, including nine aminoglycoside acetyltransferases (*aac*) present in both strain groups. Three aminoglycoside adenyltransferases (*aad* genes) were exclusive to previously characterized strains. Four aminoglycoside nucleotidyltransferases (*ant*) were detected, with ANT(3″)-II present in all strains. Aminoglycoside phosphotransferases (*aph*) were distributed across clusters 1–3 but absent in cluster 4, which exhibited the greatest ST diversity. The *armA* gene, encoding an aminoglycoside methyltransferase, was restricted to clusters 1 and 2.

*Macrolides.* Resistance genes *amvA, mphE*, and *msrE* were detected across clusters, while *ermA* and *ermC* were restricted to MIQ strains.

*Tetracyclines.* Genes *tetB, tetA, tetG,* and *tet38* were identified exclusively in MIQ. L04_100 AB54.

*Chloramphenicol.* Resistance determinants *catB8, cmlB1, cmlA,* and *floR* were detected across strains.

*Fluoroquinolones.* Resistance was associated with mutations in *gyrA* (G79C, S81L) and the presence of the *adeFGH* efflux complex in all strains. Additional efflux determinants *abaQ, emrAB-tolC*, and *emrB* were exclusive to Ab-s.3207 and Ab-s. AF-401, while *norA* was unique to MIQ. L03_100 AB54.

*Sulfonamides and Trimethoprim.* Genes *sul1* and *sul2* were widespread, whereas trimethoprim resistance mediated by *dfrC* was limited to clusters 3 and 4.

*Oxazolidinones and Lipopeptides.* Linezolid resistance was linked to mutations in 23S rRNA (G2032T), with additional variants (C2579T, G2604T) detected in three strains, suggesting recent adaptive events. Daptomycin resistance-associated mutations in *walK* and *cls* were identified exclusively in MIQ. L03.100.

### Virulome profiles

**Figure 3:**
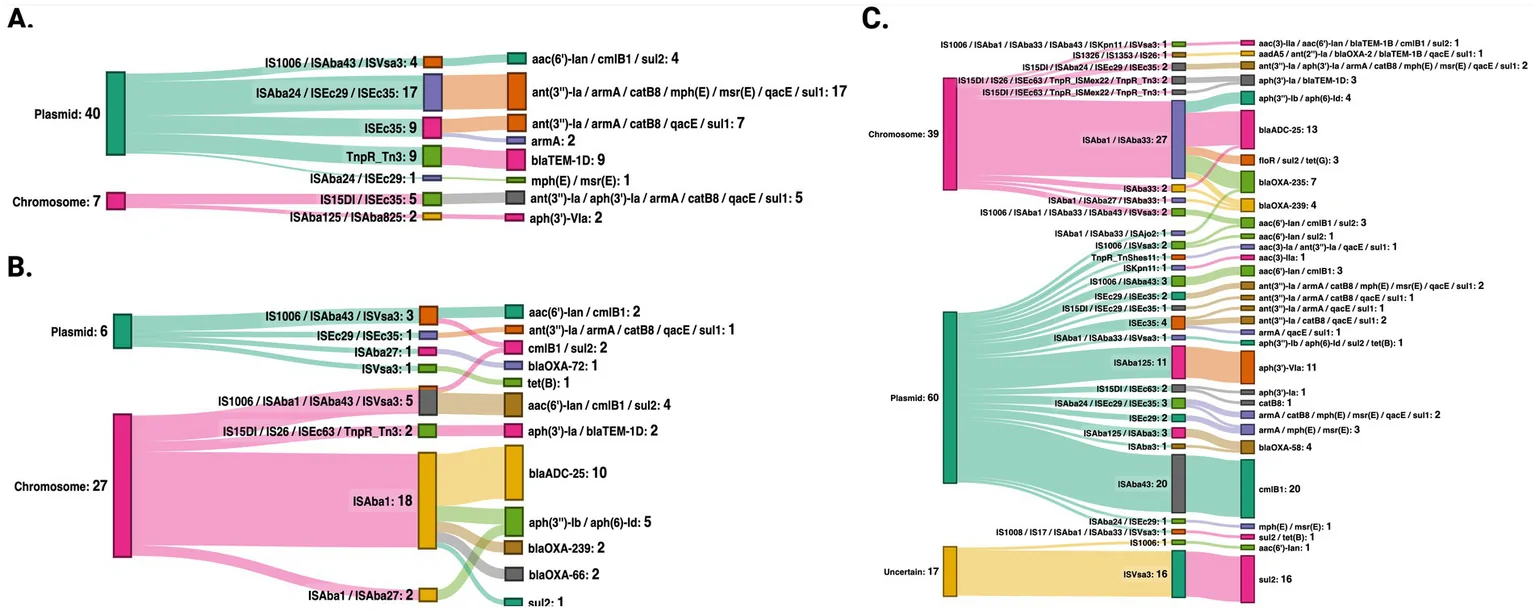
Virulome profiles. **(A)** Heatmap of virulence gene presence/absence across genomes, grouped into functional categories (adhesion, biofilm, immune evasion, metabolic adaptation, stress resistance). Rows represent strains, annotated with ST and state. Columns represent individual genes; green squares indicate presence. **(B)** Principal coordinates analysis (PCoA) based on Bray–Curtis dissimilarity of virulome profiles, showing four main clusters. Shapes distinguish MIQ and HPG genomes; colors correspond to clusters. Together, these panels demonstrate parallel evolution of virulence traits and their distribution across lineages. Created in BioRender. Alejo, A. (2026) https://BioRender.com/xadgqvw.

Virulence profiling revealed 19 distinct virulome patterns (Figure 3A), with six exclusive to MIQ strains, eight exclusive to previously characterized strains, and five shared. Post-COVID isolates carried novel biofilm and outer-membrane protein genes, suggesting enhanced persistence in hospital environments and greater potential for immune evasion. Emerging virulence determinants were observed in strains MIQ. L03_100, MIQ. MIP2, MIQ. L01_71, MIQ. L01_49, and MIQ. AB550.

Notably, virulome composition did not cluster by phylogeny or sequence type, indicating that virulence traits are more strongly shaped by horizontal gene transfer and local selection than by clonal background. This decoupling from the core genome complicates prediction of virulence potential from lineage alone. We could identify four main virulome clusters, further supported by the PcoA analysis (Figure 3B). Shared adhesion genes included *bap*, *pgaA*, *pgaB*, and *pgaC*, with notable copy number variation in *bap* (23 copies in Ab-s. H350 vs. 8 in MIQ. L01_38). Genes *pilT*, *fba*, and *tufA* were also common, while *pilA* was exclusive to MIQ. L03_100. Oxidative stress gene *katA* was present in both groups, whereas immune evasion genes *nouG* and *femA* were unique to MIQ strains. Metabolic genes *carA*, *sucC*, and *pyrAA* were exclusive to MIQ isolates, while *ilvG*, *glyA-1*, *tktA*, and *VC0243* were restricted to previously characterized strains. Additional findings included *hktE* and *rpmF* in both groups, *tilS* in MIQ. L01_71, and *glyA-1* in Ab-s. ARC6644.

**Figure 4:**
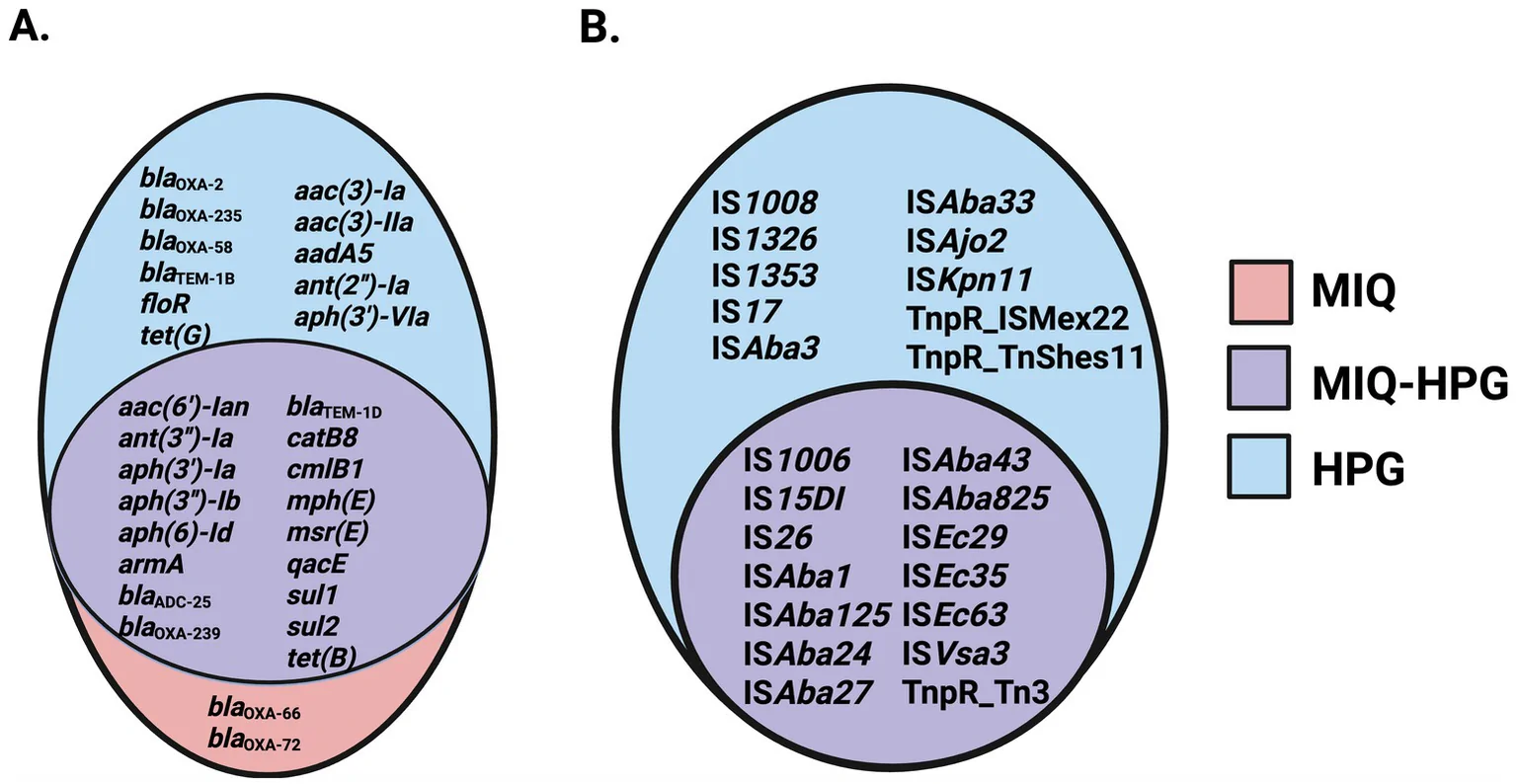
Mobilome composition. Sankey diagrams illustrating gene flow between chromosomes, plasmids and other mobile genetic elements (MGEs) in MIQ and HPG genomes **(A–C)**. Connections show associations between genomic elements, insertion sequences, transposons, and resistance genes. Created in BioRender. Alejo, A. (2026) https://BioRender.com/dt2ei3e.

These results demonstrate that virulence traits evolved in parallel with resistance, generating pathogens that are both harder to treat and more persistent in clinical settings.

### Mobilome composition

**Figure 5:**
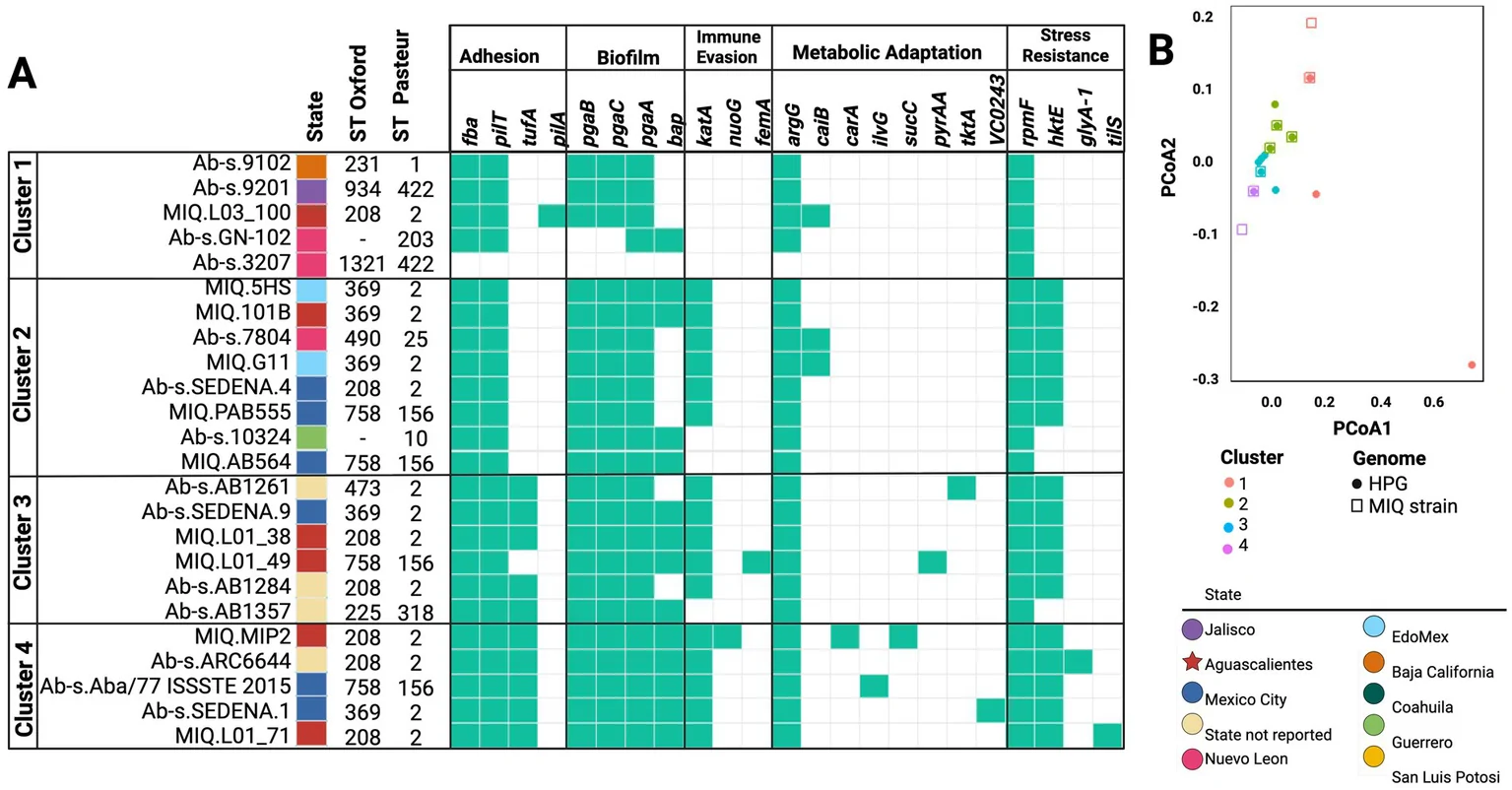
Comparative distribution of the resistance genes and MGEs. **(A)** Venn diagram comparing resistance genes among MIQ, MIQ-HPG, and HPG genomes. Unique and shared ARGs are indicated in each set. **(B)** Venn diagram comparing insertion sequences and transposons among the same groups. These comparisons reveal conserved elements, lineage-specific repertoires, and novel associations in MIQ strains. Created in BioRender.Alejo, A. (2026) https://BioRender.com/bdqbdf9.

**Figure 6:**
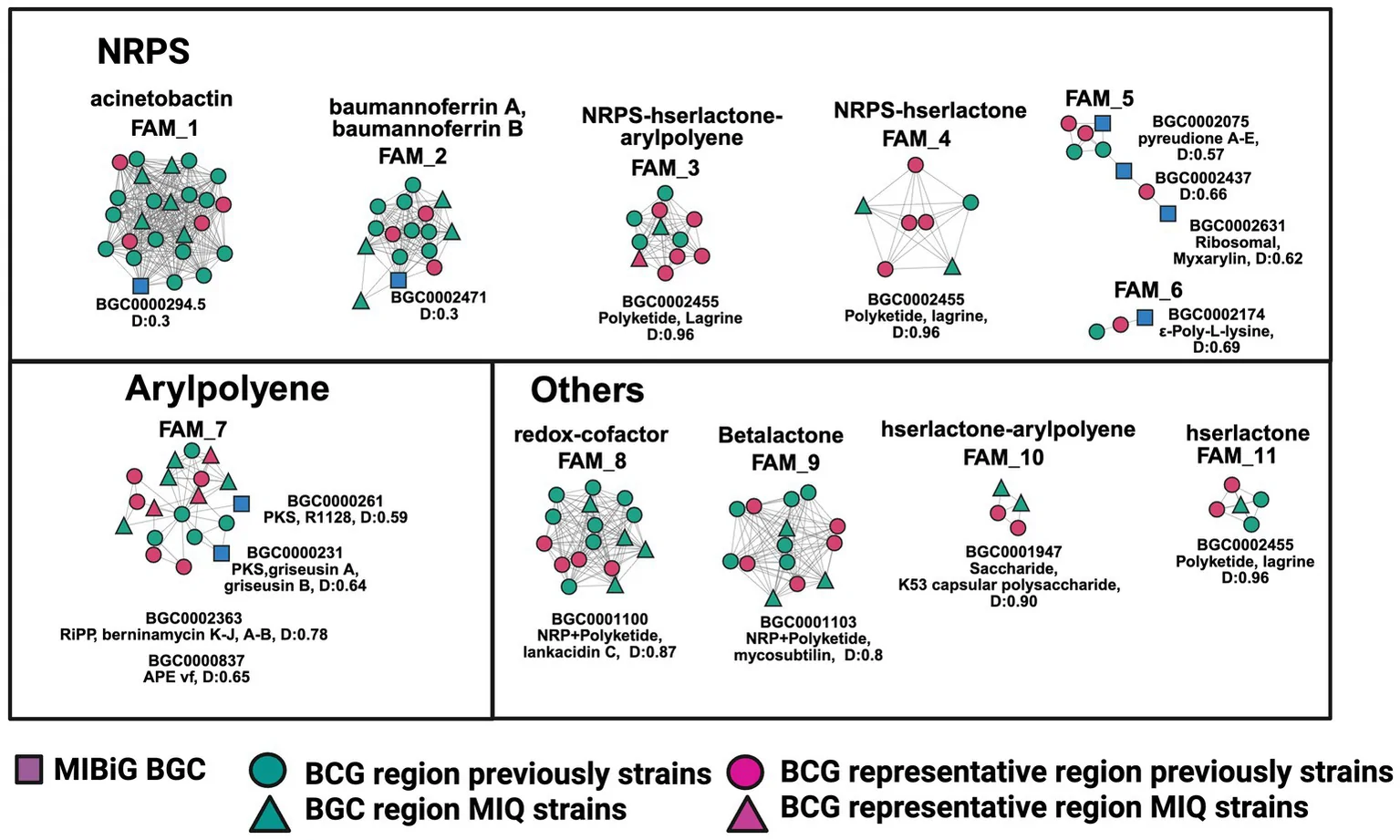
Biosynthetic gene clusters (BGCs) Similarity network of 120 unique BGCs grouped into 11 families (FAM1–FAM11) using BiG-SCAPE. Nodes represent representative regions from MIQ and HPG genomes; edges indicate similarity (distance < 0.3). Families include conserved siderophores (acinetobactin, baumannoferrin A/B) and diverse NRPS, PKS, betalactone, and hserlactone clusters. The network highlights conserved iron acquisition systems and novel BGCs with antimicrobial potential in post-COVID isolates. Created in BioRender. Alejo, A. (2026) https://BioRender.com/cfdf0ac.

Plasmids were confirmed as the principal vectors of resistance gene dissemination, with 74.2% of MGEs plasmid-associated in MIQ strains, comparable to 76.7% in previously characterized isolates (Figure 4). A total of 24 distinct mobile genetic elements (MGEs) were identified (Figure 5A) for the entire dataset. No novel MGEs were uniquely associated with MIQ strains; instead, these isolates revealed conserved MGEs linked to antimicrobial resistance gene (ARG) dissemination. In contrast, 30 different ARGs were found in genomic neighborhoods associated with MGEs, underscoring the diversity of resistance genomic context (Figure 6).

Recurrent structures included IS*1006*-associated modules carrying *sul2*, *cmlB1*, and *aac(6’)-Ian*, as well as multidrug resistance clusters involving IS*Ec29* and IS*Ec35* with *armA*, *ant(3”)-Ia*, *catB8*, *mph(E)*, *msr(E)*, *qacE*, and *sul1*. Similarly, IS*Aba1* and IS*Aba33* were consistently associated with *β*-lactamase genes such as *bla*_ADC-25_ and *bla_OXA-239_*, in agreement with previously described genomic contexts.

Special attention was given to transposon-associated recombinases, particularly *TnpR* elements. These were identified in association with β-lactamase genes, including *TnpR_Tn3* linked to *bla*_TEM-1D_ and *TnpR_TnShes11* linked to *aac(3)-Ia*, *ant(3”)-Ia*, *qacE*, and *sul1*.

We identified novel IS–ARG contexts in MIQ isolates, including *bla*_OXA72_ linked to IS*Aba27* and *bla*_OXA66_ associated with IS*Aba1*, an arrangement not observed in historic Mexican genomes in this dataset. These configurations may facilitate further mobilization of carbapenemase genes.

MIQ and MIQ-HPG genomes were enriched for IS*Aba1*-like elements and composite IS modules (e.g., IS*1006*–IS*Aba43*–IS*Vsa3*), whereas HPG-only genomes more frequently carried IS*Aba3*, IS*Aba33*, and TnpR-associated elements. This suggests a shift toward IS*Aba1*-mediated mobilization in post-COVID isolates.

### Specialized metabolism

Secondary metabolite analysis revealed conserved siderophore biosynthetic clusters across all clades, underscoring iron acquisition as a central survival strategy (Figure 6). Post-COVID isolates also carried novel biosynthetic gene clusters (BGCs) with antimicrobial potential, suggesting unexplored metabolic capacities that may influence interspecies competition and host interactions.

Using antiSMASH 7.0, we identified 1,637 genomic regions linked to specialized metabolite production across 204 genomes, with an average of eight BGCs per genome; strain Ab-s. SEDENA6 carried 11. Clustering with BiG-SCAPE 2.0 reduced redundancy, selecting 42 representative regions and yielding 120 unique BGCs grouped into 11 families (distance < 0.3).

BGC families FAM_1–FAM_6 encoded NRPS siderophores and peptides (including acinetobactin and baumannoferrin), which were conserved across clades, while FAM_7–FAM_11 comprised arylpolyene, redox-cofactor, *β*-lactone, and hserlactone clusters with higher variability, particularly in MIQ genomes. This suggests a stable iron-acquisition core with diversification in secondary metabolite repertoires.

## Discussion

Our data indicate that the COVID-19 period is associated with shifts in the genomic landscape of multidrug-resistant *A. baumannii* in Mexico.

Our MIQ collection is enriched for isolates referred to LESPA for confirmation of severe or multidrug-resistant infections, which likely inflates the apparent prevalence of carbapenemases and high ARG counts relative to unselected hospital or community isolates. We were unable to systematically compare against a contemporaneous, non-referral community dataset as this type of data is not available; however, national reports suggest lower carbapenem resistance rates in general hospitals undergoing surveillance (PUCRA reports). Therefore, our findings should be interpreted as reflecting a high-risk hospital subset rather than the full spectrum of *A. baumannii* circulating in Mexico.

By analyzing 194 genomes—including 47 newly sequenced post-pandemic MIQ isolates—we identified a population structured into two dominant clades enriched for Oxford STs 758, 208, 417, and 369, consistent with previous national reports describing Mexico’s distinct ST landscape relative to neighboring countries (2, 6). The persistence of Pasteur ST2 and ST156 confirms the long-term dominance of international clones IC2 and IC5, lineages strongly associated with carbapenem resistance and increased virulence (14–16). IC2 remains the most globally widespread lineage, whereas IC5 shows marked enrichment in Latin America, including Mexico, Brazil, and Paraguay (17), while other epidemic clones common in Asia and Europe (IC1, IC7, IC8) remain rare in Mexico (18). The detection of rare or previously unreported Oxford STs (e.g., ST1321, ST490, ST227, ST934, ST1942, ST690, ST225) suggests ongoing diversification and possible inter-hospital or inter-state transmission, emphasizing the need for coordinated regional surveillance rather than isolated hospital-level monitoring (2).

Resistome profiling revealed 128 distinct gene patterns and 44 emerging ARGs, expanding the national repertoire beyond classical carbapenemases and efflux pumps. Although these *β*-lactamase variants are not globally novel, they appear for the first time in Mexican *A. baumannii* collections, likely introduced through interspecies gene transfer mediated by mobile genetic elements (6, 19, 26). Enrichment of carbapenemase genes such as *bla*_OXA-72_ and *bla*_OXA-66_ in MIQ isolates may reflect referral bias, as hospitals preferentially submit resistant strains; thus, policymakers should interpret hospital-based genomic surveillance cautiously and complement it with community-level sampling (8, 27).

Virulome analysis showed conserved adhesion, biofilm, and immune-evasion determinants across lineages, consistent with previous studies (20), but MIQ isolates exhibited expanded metabolic-adaptation repertoires. These genes may enhance survival under nutrient limitation, oxidative stress, and antibiotic pressure—conditions typical of high-burden hospital environments—suggesting that metabolic flexibility contributes to persistence and treatment failure.

Mobilome analysis demonstrated extensive plasmid-mediated dissemination of ARGs, with notable enrichment of IS*Aba1* and IS*Aba3* in post-COVID isolates. These elements are known activators of carbapenemase expression and potent facilitators of horizontal gene transfer (19). Despite fragmented assemblies, the consistent detection of MGEs indicates active plasmid-driven ARG mobilization. Novel IS–ARG configurations such as *ISAba27*–*blaOXA-72* and *ISAba1–blaOXA-66* parallel global reports of rapidly evolving IS-mediated resistance mechanisms (21, 22) and underscore the urgency of implementing long-read sequencing in national surveillance programs (6, 27).

Specialized-metabolism analysis revealed conserved siderophores (acinetobactin, baumannoferrin) alongside novel biosynthetic gene clusters, consistent with recent global findings (10, 11). The enrichment of metabolic genes such as *argG, carA*, and *ilvC* in MIQ isolates suggests adaptation to nutrient-limited or stress-rich clinical environments, although functional validation is needed. Expanded BGC repertoires may enhance interspecies competition and persistence in polymicrobial infections, complicating treatment outcomes. This represents the first genomic survey of specialized metabolism in Mexican *A. baumannii*, highlighting both conserved virulence-linked siderophore systems and novel BGCs with potential roles in pathogenicity and antimicrobial activity.

Overall, these findings reveal a population shaped by persistent international clones, emerging local lineages, and active plasmid-mediated gene flow. For stewardship and policy, the results underscore the need for integrated, multi-hospital genomic surveillance, long-read sequencing for plasmid tracking, and coordinated regional strategies to monitor the spread of high-risk clones and mobile resistance determinants.

Together, these findings provide the first integrative genomic portrait of *A. baumannii* in Mexico before and after the COVID pandemic. They situate Mexican isolates within the global CRAb crisis, dominated by IC2 and IC5, but also reveal unique mobilome innovations and novel IS–ARG contexts. Sustained genomic surveillance, antimicrobial stewardship, and functional validation of genomic predictions are urgently needed. Without these measures, continued diversification of *A. baumannii* lineages and expansion of resistance determinants may undermine infection control efforts and therapeutic efficacy.

## Conclusion

This study provides the first integrative genomic portrait of *A. baumannii* circulating in Mexico across the pre- and post-COVID-19 periods. These conclusions are shaped by referral and sampling biases toward high-risk hospital isolates, underscoring the need for complementary community-based genomic surveillance. By analyzing 194 genomes, including 47 newly sequenced isolates, we revealed how resistance determinants, virulence factors, mobile genetic elements, and biosynthetic gene clusters converged to shape novel pathogenic lineages. The dominance of Oxford STs 758, 208, 417, and 369 highlights the persistence of internationally recognized clones, while the emergence of rare sequence types signals ongoing local diversification.

Beyond these core findings, several additional observations carry important public health relevance. Phylogenetic analyses demonstrated geographic mixing of clades across Jalisco, Aguascalientes, and Mexico City, suggesting inter-hospital or inter-state transmission routes that demand coordinated regional surveillance rather than isolated hospital-level monitoring. Resistome profiling revealed referral bias toward carbapenemase-positive isolates, underscoring the need to complement hospital-based genomic surveillance with community-level sampling to avoid overestimating resistance prevalence. Virulome analysis showed enrichment of metabolic adaptation genes in MIQ strains, pointing to enhanced resilience that may facilitate persistence in hospital environments and increase outbreak risk. Mobilome profiling identified enrichment of IS*AbA1* and IS*AbA3* elements in post-COVID isolates, highlighting ongoing plasmid-mediated dissemination of carbapenemase genes and the urgency of incorporating long-read sequencing into surveillance frameworks. Finally, biosynthetic gene cluster analyses revealed expanded repertoires in MIQ genomes, suggesting adaptive metabolic diversification that may enhance ecological fitness and complicate treatment outcomes.

Taken together, these findings emphasize that *A. baumannii* in Mexico is not only evolving through classical resistance mechanisms but also through geographic mixing, referral biases, virulome expansion, mobilome diversity, and specialized metabolism. Addressing these challenges requires integrated genomic surveillance, functional validation of adaptation signals, and regionally coordinated infection control strategies. Without such measures, the continued diversification of *A. baumannii* lineages and expansion of resistance determinants may undermine therapeutic efficacy and infection control efforts in the post-COVID era.

## Data Availability

The original contributions presented in the study are included in the article/Supplementary material, further inquiries can be directed to the corresponding author/s.

## Footnotes

1
http://pubmlst.org/abaumannii

## Supplementary material

Supplementary Table 1 Metadata of *Acinetobacter baumannii* isolates collected from hospitals, environmental sources, and reference strains across Mexico. The table includes GenBank accession numbers, genome identifiers, collection year, hospital/state of origin, sequence type (ST) assignments (Pasteur and Oxford schemes), patient demographics, comorbidity status, COVID-19 test/vaccine information, and clinical outcomes.

Supplementary Table 2 Distribution of antimicrobial resistance (AMR) gene families across *A. baumannii* isolates. Gene presence/absence is reported per genome, grouped by functional categories (aminoglycoside, β-lactam, macrolide, sulfonamide, tetracycline, etc.), enabling comparative analysis of resistance determinants between MIQ and historic strains.

Supplementary Table 3 Virulence gene distribution across *A. baumannii* isolates. The table details the presence of genes associated with adhesion, biofilm formation, iron acquisition, secretion systems, and other virulence traits, highlighting variability across clinical and environmental strains.

Supplementary Table 4 Mobile genetic elements (MGEs) identified in *A. baumannii* genomes. The table lists ARG-associated MGEs, including plasmids, integrons, transposons, insertion sequences (IS), genomic islands (GI), integrative conjugative elements (ICE), and prophages. Coordinates and replicon details are provided to illustrate the genomic context of resistance and virulence determinants.

Supplementary Table 5 Biosynthetic gene clusters (BGCs) identified in MIQ and historic *A. baumannii* strains. The table reports cluster types (e.g., siderophores, non-ribosomal peptide synthetases, polyketides), genomic coordinates, and predicted functions, enabling comparative analysis of secondary metabolite potential across isolates.

Supplementary Methods Detailed bioinformatics pipeline for resistome, virulome, mobilome, and BGC analyses.
